# Natural Building Materials for Interior Fitting and Refurbishment—What about Indoor Emissions?

**DOI:** 10.3390/ma14010234

**Published:** 2021-01-05

**Authors:** Matthias Richter, Wolfgang Horn, Elevtheria Juritsch, Andrea Klinge, Leon Radeljic, Oliver Jann

**Affiliations:** 1 Materials and Air Pollutants Division, Bundesanstalt für Materialforschung und -prüfung (BAM), Unter den Eichen 44-46, 12203 Berlin, Germany; wolfgang.horn@bam.de (W.H.); ria.juritsch@bam.de (E.J.); oliver.jann@bam.de (O.J.); 2 ZRS Architekten Ingenieure, Schlesische Straße 26, 10997 Berlin, Germany; radeljic@zrs.berlin

**Keywords:** bio-based insulation, earthen building materials, volatile organic compounds, semi-volatile organic compounds, formaldehyde, radon

## Abstract

Indoor air quality can be adversely affected by emissions from building materials, consequently having a negative impact on human health and well-being. In this study, more than 30 natural building materials (earth dry boards and plasters, bio-based insulation materials, and boards made of wood, flax, reed, straw, etc.) used for interior works were investigated as to their emissions of (semi-)volatile organic compounds ((S)VOC), formaldehyde, and radon. The study focused on the emissions from complete wall build-ups as they can be used for internal partition walls and the internal insulation of external walls. Test chambers were designed, allowing the compounds to release only from the surface of the material facing indoors under testing parameters that were chosen to simulate model room conditions. The emission test results were evaluated using the AgBB evaluation scheme, a procedure for the health-related evaluation of construction products and currently applied for the approval of specific groups of building materials in Germany. Seventeen out of 19 sample build-ups tested in this study would have passed this scheme since they generally proved to be low-emitting and although the combined emissions of multiple materials were tested, 50% of the measurements could be terminated before half of the total testing time.

## 1. Introduction

Indoor environment has a significant influence on human health and our perception of well-being. Knowledge of indoor air quality, its significance for our health, and the factors that cause poor air quality are crucial to enable relevant stakeholders—including building owners, developers, users, and occupants—to maintain clean indoor air. Emissions from construction products constitute a significant source of indoor pollution and result, under certain environmental and occupational conditions, in sensory irritation and health complaints [1,2,3,4]. This represents a symptom of the fact that in western countries, more than 80% of the time is spent indoors [5,6]. A wide range of volatile organic compounds (VOCs), semi-volatile organic compounds (SVOCs), and carbonyl compounds (including formaldehyde) can be released from construction materials. Emissions concentrations become further elevated in new or refurbished buildings [7], where the rate of air exchange with fresh ambient air may be limited due to improved energy saving aspects [8], which is one of the main reasons for poor indoor air quality. The perpetuation of an air exchange rate of 0.5 h^−1^ is commonly recommended from a hygienic point of view [9]. In Europe, all new buildings should be nearly zero-energy buildings by the end of 2020 [10], which means that air exchange rates, and consequently indoor air quality levels, are set to decrease further. With a view to countering this trend, it is reasonable to suggest that building materials used indoors should be as low-emitting as possible. Furthermore, they should have the capacity to buffer moisture and anthropogenic indoor pollutants (e.g., from cooking, heating, smoking, etc.), as well as being recyclable and sustainable in terms of embodied energy.

Natural building materials, such as wood, cellulose, and earth, meet these criteria. They can easily be dismantled and reused or composted and are therefore the first choice to reduce environmental footprint and life-cycle costs. Moreover, they have excellent hygrothermal and acoustic properties, and show higher adsorptive capacities related to VOCs than other building materials [11,12].

Since the use of modern bio-based insulation materials is not widespread, only a few investigations on material emissions have been published [13]. In the study presented here, the emissions of formaldehyde, VOCs, SVOCs, and radon from different combinations of more than 30 natural materials (earthen dry boards and plasters, bio-based insulation materials, and boards made of wood, flax, reed, straw, etc.) underwent emission testing in specially designed test chambers. In standardised test procedures, it is normal to investigate materials individually. The results from individualised emission rate testing helps derive potential VOC indoor concentrations but does not take into account the combination effects arising from different source’s emissions. The test series were arranged such that either single materials (first and foremost earth plasters) or complete wall assemblies in different combinations were tested. An evaluation of the results according to the AgBB scheme [9] was carried out to decide if the materials or material combinations are suitable for indoor use.

## 2. Materials and Methods

### 2.1. Materials

In Table 1 and Table 2, the tested materials as well as the different material combinations tested for emissions are listed. The combinations are selected as to structural-physical considerations. Based on a literature study (scientific journals, conference proceedings, research reports), building materials suitable for flat separation walls, internal partition walls, and internal insulation of external walls have been identified and selected. Special emphasis was placed on those earth plasters that demonstrate an increased moisture buffer capacity as well as on wood fibre boards, not only because they are good moisture adsorbers, but also since they can prevent against overheating in summer and contribute to improved room acoustics. In addition, innovative construction materials, e.g., strawboards or sandwich boards made out of earth plaster and wood fibre boards that bear the potential to speed up the construction process, have been selected: in many cases, earthen materials (a9–a11) with different additives (b1–b4) to enhance their functionality are used for wall finishing. It is well known that earthen materials contribute to a healthy room climate [14,15]. Furthermore, it is a sustainable building material that can be used as a structural [16] or non-structural material (e.g., in plasters) [17]. Once at the end of its service life, earthen materials can be recovered and reused without loss of performance [18]. The capacity for moisture buffering of earthen and wooden materials is four to five times higher than that of conventional building materials [19]. In order to increase this effect, the earth mortars were modified with silica aerogel material Quartzene^®^. Aerogels have not only low thermal conductivities, but due to their high specific surface area, they provide excellent sorption properties. The optimisation of these materials was part of another investigation and therefore sample build-ups finished with earth plaster containing different amounts of aerogel were also tested for emissions.

Soil-based materials like clay contain naturally occurring radionuclides, such as U-238, Th-232, and K-40 [20], and tend to emit the radioactive noble gas radon (Rn-222) [21], which is classified by the World Health Organization (WHO) to be the second most common cause for lung cancer after smoking [22]. In order to accommodate this fact, radon was added to the emitters of interest.

Besides earthen materials, dry boards and insulation materials made of renewable resources including cellulose, flax, reed, straw, and wood were tested. Expected emissions from wood-based materials are terpenes, acids, and aldehydes. Though wood-based materials are often perceived as pleasantly odorous, their emissions can also cause allergies, e.g., irritation of the mucous membrane and skin [23]. Numerous studies have shown that terpenes emitted from wood can combine naturally with ozone in the atmosphere to produce irritants which could be responsible for eye and airway complaints [4,24,25]. Besides hexanal, one of the most often identified wood-based aldehydes, formaldehyde, can also be emitted depending on the type of timber [26]. Formaldehyde is also an ingredient in synthetic resins used in the production of mineral wool [27,28]. Two types of mineral wool (f1, f2) with different production technologies were tested.

In this study, since the final emissions of the combined materials were of interest, for most of the single materials, no emission tests were carried out, except for the samples 1–6 (Table 2). For such data, it is referred to measurements carried out by Hofbauer, W., Krueger, N. [13].

### 2.2. Sample Preparation

**Figure 1:**
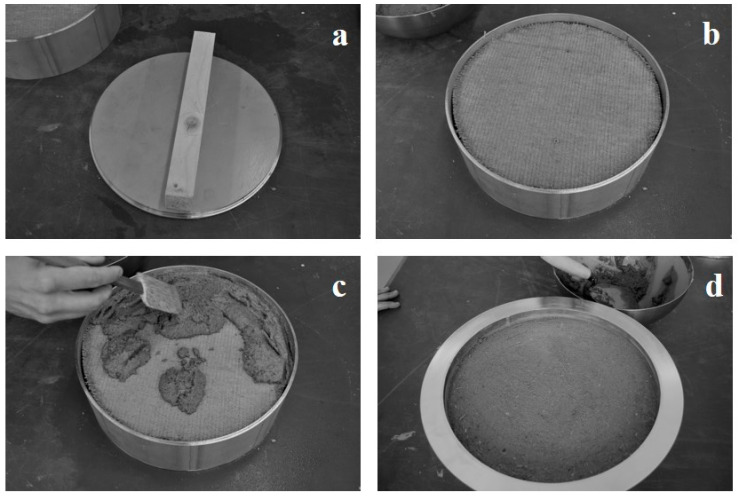
Sample installation into sample intake: (**a**) timber stud, (**b**) mounted earth dry board (wood fibre insulation below), (**c**) earth plaster final coat, (**d**) mounting of flanged section ring for installation into test chamber.

All sample materials were installed in sample intakes completely made of stainless steel, as depicted in Figure 1. Each one consists of several parts designed to fit into each other and which can be arranged to different heights, aiming to produce test specimens of varying thickness. The bottom is made of a plate with a furrow at its edge to allow the stacking of various sections (concentric rings) with an inner diameter of 314 mm, which were produced from a commercially available tube. Abutting edges were sealed with self-adhesive aluminium tape to ensure air tightness. All sample materials were built up layer by layer into the intakes, taking care that they exactly fitted the dimensions of the rings. The last layer was applied flush with the top edge of the last ring and was in most cases a plaster. The plasters were applied with the manufacturer-recommended water content using a plastering trowel.

Solid samples were prepared by cutting a circular piece from the centre area of the board. Build-ups made of natural building materials and finished with earth render were pre-conditioned at 23 °C and a relative humidity (RH) of 50% in order to prevent the samples elevating the RH in the emission test chamber above the required test conditions during testing as described in Section 2.3. As a rule of thumb, earthen plasters have reached their state of “intended use” after a drying time of about 14 days. Hence, this period was chosen as the maximum possible conditioning time because there are no product standards available yet specifying requirements for emissions testing.

The described sample preparation procedure is seen as beneficial since no additional sealing of edges is required. The sealing is represented by the wall of the concentric rings and it is ensured that only the surface normally facing indoors is exposed. Thus, the impact of all combined materials on the indoor air quality can be accounted for.

### 2.3. Emissions Testing

**Figure 2:**
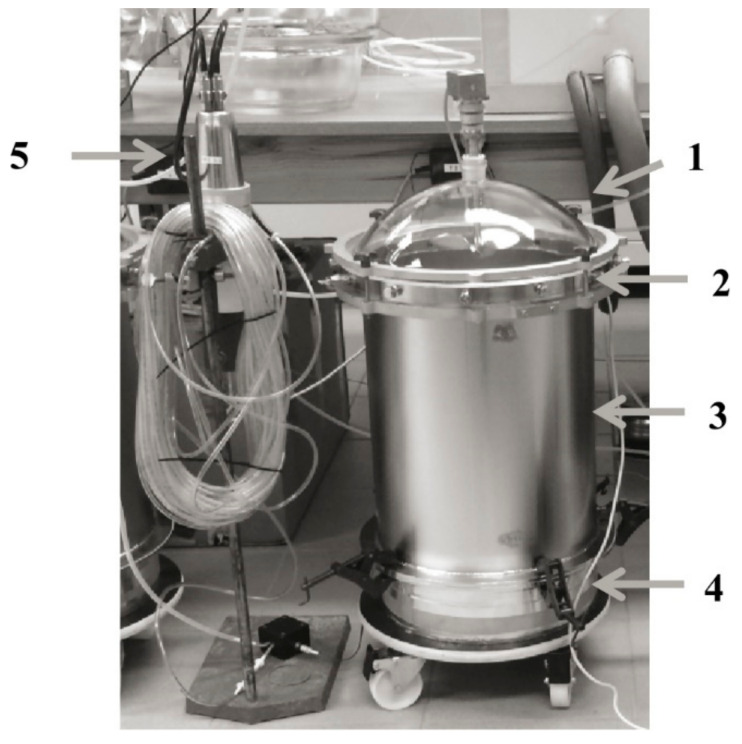
Emission test chamber assembly with Lucas scintillation cell for radon measurement: (**1**) glass lid with agitator, (**2**) connection ring, (**3**) hollow cylinder, (**4**) sample intake, (**5**) Lucas cell.

The emission tests were carried out in specially designed test chambers as depicted in Figure 2. Mounted onto the sample intake (4) is the actual test chamber comprising a cylinder with 420 mm in height (3), a stainless steel connection ring (2) with ports for air supply and sampling, and a glass lid (1) equipped with an agitator for the homogenisation of the test chamber air. The overall chamber volume above the surface of the test specimen is 38.5 L. The tests were conducted according to the requirements of EN 16516 [29] at a temperature of (23 ± 1) °C and a relative humidity (RH) of (50 ± 5)%.

Radon exhalation was performed in parallel on the basis of the procedure published by Hofmann et al. [30,31,32].

The test chambers were operated dynamically by applying an air change rate *n*; *n* is defined as the ratio of air volume fed into the test chamber to the free volume of the test chamber and should be set to 0.5 h^−1^ in order to simulate normal indoor air conditions. Representativeness with regards to the intended use of the test samples is assured by applying a product-loading factor L of 1.0 m^2^/m^3^ for wall materials. L is defined as the ratio of the exposed area of the test specimen and the volume of the test facility. The ratio of *n* and L makes the area specific air flow rate q (0.5 m^3^/m^2^h). Considering an exposed surface area of 0.025 m^2^, the air flow through the test chamber was adjusted to 0.62 L/h.

Emission tests for the evaluation of construction products normally last 28 days. After this period, either steady-state emissions have been reached or the decay of emissions has slowed down significantly. If such a situation was achieved before the end of the testing period, the test was terminated, which was in some cases after the 10th sampling day.

### 2.4. Sampling and Analysis

Air sampling and analysis were carried out according to ISO 16000-3 [33] and ISO 16000-6 [34] at days 3, 7, 10, 14, and 28 after sample installation in the test chamber. Sampling took place at 23 °C and the analysis immediately after sampling.

Cartridges filled with the adsorbent 2,4-Dinitrophenylhydrazine (DNPH) (Supelco, Bellefonte, PA, USA) were used for the determination of carbonyl compounds (aldehydes and ketones), particularly formaldehyde; while for the determination of VOCs and SVOCs, glass tubes filled with the adsorbent Tenax TA^®^ (Supelco, Bellefonte, PA, USA) were used. The sampling volume on DNPH cartridges was 30 L (60 min at a sampling flow rate of 500 mL/min). Afterwards, they were eluted with a mixture of acetonitrile and water (volume ratio 4:1). This solution was then analysed using high pressure liquid chromatography equipped with a diode array detector (HPLC-DAD) on a ZORBAX Eclipse XDB-C18 column (4.6 mm × 150 mm, 5 µm, Agilent, Santa Clara, CA, USA) with methanol and water as mobile phase.

For Tenax sampling, 1 L was taken at 100 mL/min for 10 min, followed by thermal desorption of the tubes and analysis using gas chromatography on a slightly polar column (Rxi^®^-5 ms, 30 m × 0.25 mm × 1.0 µm, Restek, Bellefonte, PA, USA) and with a mass selective detector (GC-MS) (Agilent, Santa Clara, CA, USA). In general, duplicate sampling was carried out. All identifiable VOCs that can be found on the LCI-list (cf. Section 2.5) were quantified by using their individual response factor. Compounds that are not listed or show a mass spectrum that cannot specifically be assigned to a certain compound were quantified by use of the response factor for toluene (toluene equivalent). The blank value of the adsorbent tube was determined by analysis of the unloaded tube prior to sampling and subtracted. SVOCs were quantified by integrating the chromatogram between the elution range of hexadecane and docosane with toluene equivalents, giving the sum parameter ΣSVOC.

**Figure 3:**
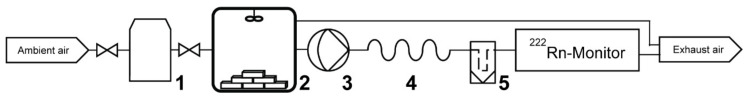
Sampling procedure with thoron delay line: (**1**) air supply system, (**2**) loaded emission test chamber, (**3**) sampling pump, (**4**) delay line, (**5**) radon progeny filter [32].

Radon (Rn-222) measurement was performed using a calibrated, self-made Lucas scintillation cell. The cell itself is a small chamber of about 250 mL, whose inside walls are coated with silver-activated zinc sulphide, ZnS(Ag). In use, a filtered sample of chamber air continuously enters the cylinder. The Lucas cell is placed on a photomultiplier tube (PMT) inside a light-tight enclosure. Alpha particles emitted by radon and radon decay products strike the ZnS(Ag) phosphor, which emits light pulses that are amplified by the PMT and then counted by an alpha counter (Ortec Digibase) (Ortec, Oak Ridge, TN, USA). For more information on the measurement procedure, refer to Quindos-Poncela, L.S., Fernandez, P.L. [35]. A shortcoming of the procedure is the non-selectivity towards Rn-222 and its isotope Rn-220 (thoron). In order to be able to discriminate both, chamber air is led through a PVC hose (delay line) before entering the measuring cell. The tube is as long as three half-lives of the short-lived Rn-220 (λ_Rn-220_ = 55 s, λ_Rn-222_ = 3.8 d), making 24 m at an inner diameter of 4 mm and a continuous sampling flow of 110 mL/min (Figure 3). This procedure was described by Hofmann, M., Richter, M. [32].

### 2.5. Evaluation of Measurement Results

The measurement results were evaluated against the German AgBB scheme [9]. The procedure is based on the analysis of test chamber air sampled on at least the 3rd and 28th day after loading. The following parameters are monitored:

- TVOC (total VOC): sum of the concentration of all individual substances with concentrations equal to or greater than 5 μg/m^3^ within the retention range C6–C16.
- ΣSVOC: sum of the concentration of all individual substances with concentrations equal to or greater than 5 μg/m^3^ within the retention range > C16–C22.
- Carcinogenic substances belonging to EU categories 1 and 2 or EU categories 1A and 1B.
- Assessable compounds: all VOCs with an LCI value; those compounds are listed in the appendix of the scheme; R ≤ 1.
- Non-assessable compounds: sum of VOC with, which cannot be identified, or do not have an LCI value.

The so-called R-value is based on the results of the assessable compounds on the 28th sampling day, or earlier in the case that the test can be prematurely terminated. It is a sum parameter calculated according to Equation (1) and may not be greater than 1.

*R_i_* = ∑*_i_*(*c_i_*/*LCI_i_*), (1)

with *c_i_* as the chamber air concentration of compound *i* and *LCI_i_* as the Lowest Concentration of Interest of compound *i* as listed in the annex of the AgBB evaluation scheme [9]. This value must not be exceeded by any of the listed analytes.

For the evaluation of the radon exhalation, no criteria are actually defined. European council directive 2013/59/EURATOM [36] sets limits for a maximum indoor radon concentration in new and existing buildings (300 Bq/m^3^), but how much the contribution of the building material can be is not yet defined. In Germany, the Federal Office for Radiation Protection (BfS) proposed to limit the total indoor radon concentration to 100 Bq/m^3^, whereby building materials should contribute at most 20 Bq/m^3^ [37], taking into account that the main source of radon is from soil. This criterion was adopted for the evaluation of the tested materials in this study.

## 3. Results and Discussion

### 3.1. (S)VOC Emissions and Formaldehyde

In general, with all tested materials and material combinations, low to very low indoor formaldehyde, VOC, and SVOC concentrations were determined. Table 3 gives an overview of the TVOC and ΣSVOC for all sample build-ups at the last sampling day, which was in most cases the 28th. However, in some cases, the testing was terminated after the 10th day, when the values determined were less than half the requirements for the 28th-day values and no significant increase in the concentration of individual substances was observed in comparison to the measurement on day 3.

**Figure 4:**
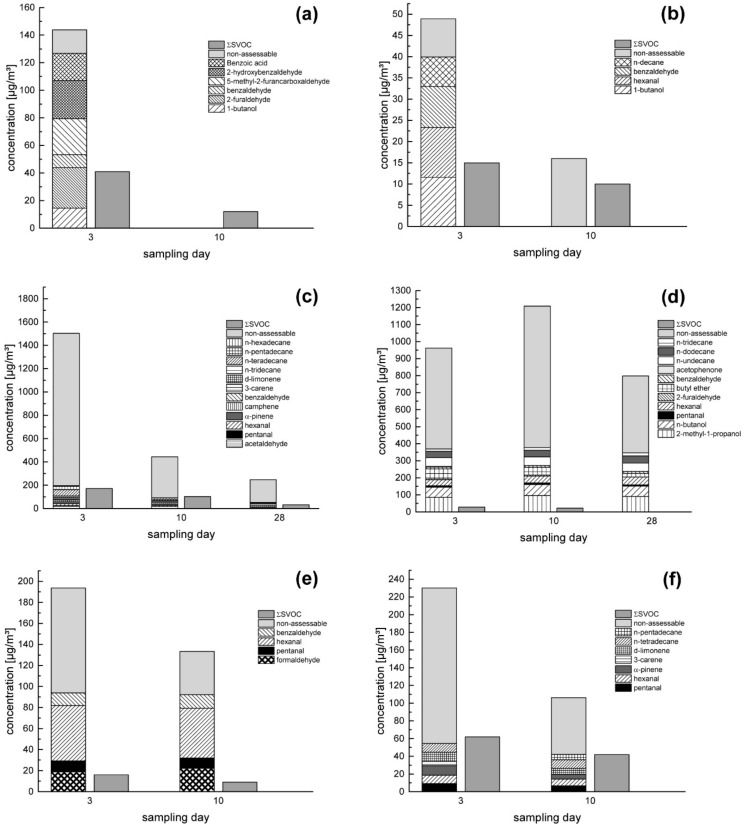
Measured volatile organic compounds (VOC) and sum parameter of semi-volatile organic compound (ΣSVOC) concentrations of six selected samples showing single VOC concentrations: samples no. 7 (**a**), no. 8 (**b**), no.11 (**c**), no. 13 (**d**), no. 14 (**e**), no. 15 (**f**).

According to the AgBB scheme, the value shall not exceed 1000 µg/m^3^ for the TVOC and 100 µg/m^3^ for the ΣSVOC on the 28th day after sample installation into the emission test chamber. The TVOC is furthermore divided into assessable and non-assessable compounds. The former are taken into account when calculating the R-value, the latter may not exceed 100 µg/m^3^ after 28 days or 50 µg/m^3^ after 10 days. Due to this criterion, samples no. 11 and 13 would not have passed the evaluation criteria and would therefore not be considered suitable for indoor use. However, due to the fact that the AgBB criteria were developed for individual building materials, it is supposed that for the analysis of wall systems, a different set of criteria would be more appropriate in further studies. In Figure 4, six detailed results of samples finished with earthen and non-earthen coatings are exemplarily depicted. All assessable and quantified VOCs are presented in the stacked bar charts, added to the non-assessable compounds, thus resulting in the TVOC. For all other results, please refer to the Supplementary Materials section (Figure S1).

All measurements were carried out in the same way as simulating conditions in a reference room, as described in Section 2.3. Therefore, all values are given in mass concentrations in µg/m^3^. It can be assumed that those concentrations would result from installing the sample materials in an equivalent environment.

With the exception of samples no. 13 and 14, the expected decrease of the concentrations over time can be observed. Sample no. 13 showed a very constant emission profile over the whole 28 days, with a significant increase of non-assessable compounds on day 10. This build-up was the one with the highest emissions over the whole testing period. Sample no. 14 emitted compounds at a much lower level, with hexanal as main VOC, presumably released by the wood fibre part of the sandwich board with a flax core. The course of the emission profile of sample no. 14 was not observed until the 28th sampling day, since the termination criterion was reached at day 10 (cf. Table 3).

With the exception of samples no. 7 and 13, where 2-furaldehyde emissions were found on day 3 in concentrations of 21 and 6 µg/m^3^, respectively, no further carcinogenic compounds were identified in any of the samples. 2-furaldehyde emissions had completely disappeared at day 10 in both cases.

Sample no. 8 showed medium initial concentrations, which rapidly decreased until day 10 when the tests were aborted. In this case, the increase of the non-assessable compounds from day 3 to day 10 is noticeable. Sample no. 15 showed only a moderate decrease of VOC concentrations, with a relatively high portion of non-assessable compounds at the beginning. The testing was aborted after day 10, although the sum of the non-assessable compounds was 64 µg/m^3^, slightly higher than the termination criterion of 50 µg/m^3^. However, the decrease between day 3 and day 10 was relatively fast and the LCI of the relevant compounds was low.

In most of the cases, the high proportion of not significantly identifiable or assessable VOCs relative to the identified ones is remarkable. One reason for this could be that in the inorganic earthen materials, a relatively small amount of organic material is present, either naturally or artificially. This undefined mixture could lead to an accumulation of different organic compounds with individually low concentrations. The relatively small amount of non-assessable VOCs in samples no. 7 and 8 might underpin this assumption as they were not coated with earth plasters.

The values for ΣSVOC were at an overall low level. At the beginning, samples no. 5 and 19 showed comparatively high SVOC releases that decreased rapidly until the last sampling day. Although SVOC concentrations may increase over time [38], there was no indication that this would occur during the testing, so the experiments were terminated before the 28th sampling day.

The sample build-ups containing wood-based insulation performed surprisingly well. In their study, Hofbauer, W., Krueger, N. [13] found that, in two of three investigated wooden samples, considerably high values for acetic acid (346 and 724 µg/m^3^, LCI-value: 1250 µg/m^3^) were present. In the third wooden material, they found a 2-furaldehyde concentration of 84 µg/m^3^ (LCI-value: 20 µg/m^3^). Other typical wood emissions, such as terpenes and aldehydes, particularly formaldehyde, were not reported by the authors or were found in negligible amounts. From all sample build-ups investigated in the current study that contained wood fibre insulations, i.e., samples no. 9, 10, 13, 14, 15, and 18, acetic acid and terpenes were found in negligible amounts on the 28th sampling day; aldehydes, e.g., hexanal, were found in concentrations between 7 and 48 µg/m^3^, which, compared to its LCI-value of 900 µg/m^3^, is very low as well. This difference could either be explained by low-emission properties in general or by a possible buffering effect from the earthen materials mounted in front of the insulation. Since clay has a significant sorptive capacity for water (e.g., McGregor, F., Heath, A. [14]), it seems that this is also true for VOCs, particularly the polar ones.

Formaldehyde was also measured in negligible concentrations ranging between 4 µg/m^3^ (samples no. 15, 16, and 19), 13 µg/m^3^ (sample no. 12), and 22 µg/m^3^ (sample no. 14). From sample no. 7 (glass wool insulation), no formaldehyde was emitted. The LCI value is set to 100 µg/m^3^.

When comparing the results of all measurements with the AgBB criteria (Table 3), all build-ups investigated in this study, with the exception of samples no. 11 and 13, would have passed the test. The reason in both cases is the same. The portion of non-assessable VOCs may not be higher than 10% of the TVOC, which was exceeded in both cases, most significantly by sample no. 13. The reason for these high amounts cannot be satisfactorily clarified, since measurements on individual materials were not planned in the study for time reasons. However, due to the fact that no harmful substances had been found in both cases on the 28th sampling day, there is no evidence that those build-ups would have any undesirable impact on occupants. This especially applies for sample no. 11, where indication for a further decrease of the emissions was given. Conversely, in sample no. 13, this trend was not observed. Particularly in buildings with air exchange rates lower than the 0.5 h^−1^ used in this study, the overall concentration level could be too high in the end.

### 3.2. Radon Exhalation

**Figure 5:**
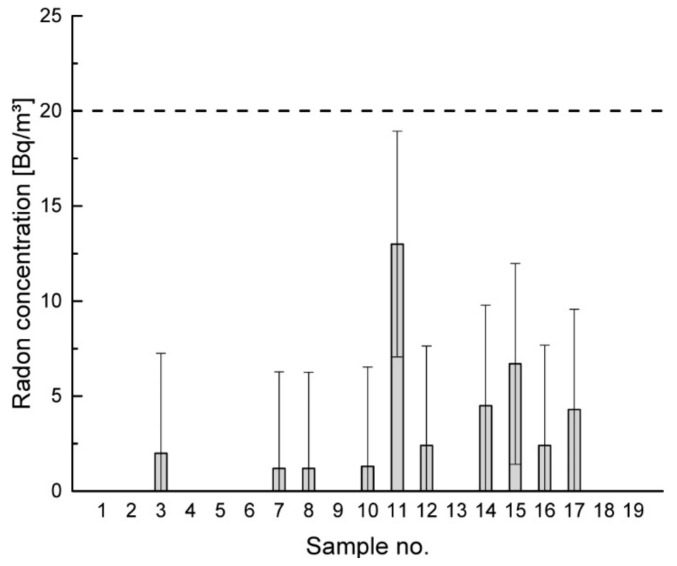
Measured radon concentrations of samples foremostly containing earthen materials.

In Figure 5, the results of the radon measurements are presented. The concentrations ranging between 2 and 13 Bq/m^3^ were considerably lower than the recommended maximum contribution of building materials to the indoor radon concentration of 20 Bq/m^3^ (dashed line) that corresponds to an annual dose of about 1 mSv. In the diagram in Figure 5, the expanded measurement uncertainty is shown (k = 2). It is ranging between 5.0 and 5.3 Bq/m^3^, indicating that the concentrations are very close to, or in some cases below, the limit of quantification, which is about 2 Bq/m^3^. The expectable indoor radon concentration is therefore almost negligible. In their study, Richter, M., Jann, O. [39] found the correlation between installed mass of radon-exhaling building materials and radon concentration; therefore, those low concentrations may result from the low amount of earthen material proportional to the complete wall build-up. Consequently, higher indoor radon concentrations are expected when higher amounts of earthen material are applied, which can be observed for sample no. 11, almost completely consisting of earth, however, still well below the recommended threshold.

## 4. Conclusions

This study pursued two aims, the evaluation of natural building materials in terms of their potential impact on indoor air quality, as well as the development of a test procedure for the reliable measurement of composite materials under standardised conditions. It revealed a good applicability of the new test chamber design in accordance with the established emission test chamber standard ISO 16000-9. Furthermore, all tested natural building materials were found uncritical with respect to their emission properties and could be installed in buildings in almost any combinations. The results of previous studies, which attested to the low emissions generated by insulation materials made from renewable raw materials, were confirmed. However, two sample build-ups did not meet the requirements of the AgBB evaluation scheme (samples no. 11 and 13) due to the emission of non-assessable compounds. However, it is noticed that in the reported work, the combined emissions from the different build-up layers were investigated. In standard tests, only the emissions from single materials are taken into consideration, disregarding the combined effect. For such investigations, an adaptation of the guideline values would be necessary. It was furthermore shown that the radon exhalation from the earthen materials was consistently below the recommended threshold of 20 Bq/m^3^. The beneficial influence of these materials on the indoor environment in terms of being an active agent to regulate indoor RH and prospectively to reduce the presence of indoor pollutants helps to produce a healthier environment, prevailing over any potential health risks, as have been investigated and published in the past.

## Figures and Tables

**Table 1:** Matrix of materials that are combined (combinations see Table 2).

	Type	Material	Thickness (mm)	Identifier
Coating	Paint	Dispersion type	0.25	a1
		Casein	-	a2
		Earth basis	-	a3
	Flour	Marble	-	a4
	Chalk	-	-	a5
	Primer	Casein	-	a6
	Joint filler	Gypsum	0.25	a7
	Deep penetrating primer	-	-	a8
	System compatible filler	Earth basis	3.0	a9
	Plaster	Earth coarse grained (final coat)	s. Table 2	a10
		Earth coarse grained (base coat)	s. Table 2	a11
		Earth fine grained (final coat)	s. Table 2	a12
Additives	-	Straw	-	b1
	-	Cellulose	-	b2
	-	Aerogel	-	b3
	-	Naturally coloured clay (red)	-	b4
Adhesives	-	Earth	4.0	c1
	System compatible	-	1.0	c2
Reinforcement	Fibre	Flax	0.5	d1
	Fibre	Glass	0.5	d2
Panels	Dry board	Gypsum	12.5	e1
		Earth	15.0	e2
			20.0	e3
		Straw	60.0	e4
	Fibre board	Gypsum	18.0	e5
		Wood	20.0	e6
		Wood fibre/flax core	70.0	e7
Insulation	Yellow (standard)	Mineral wool	60.0	f1
	Brown (eco technology)		40.0	f2
	Blocks	Wood fibre	40.0	f3
			60.0	f4
		Wood fibre (conifer)/cellulose core	120.0	f5
		Reed	100.0	f6
	Dry board	Calcium silicate	30.0	f7
Studs		Wood	60.0	g1
		Sheet steel	50.0	g2
Other	Blocks	Earth	115.0	h1
		Autoclaved aerated concrete	100.0	h2

**Table 2:** Matrix of sample build-ups. Material identifiers (Reference to Table 1) are listed in the order of their installation in the sample, starting with the side facing the interior.

	No.	Name	Materials	Thickness (mm)	Comment
Renders	1	Earthen render (final coat) A	a10/b1	5	benchmark
	2	Earthen render (final coat) B	a10/b1 *(4 parts)*, b3 *(1 part)*	5	new development
	3	Earthen render (base coat) A	a11/b1	10	benchmark
	4	Earthen render (base coat) B	a11/b1 *(4 parts)*, b3 *(1 part)*	10	new development
	5	Earthen render (final coat) C	a12/b2	3	benchmark
	6	Earthen render (final coat) D	a12/b2 *(4 parts)*, b3 *(1 part)*	3	new development
Internal partition walls	7	Dry lining wall—gypsum plaster board	a1, a7, a6, e1, f1, g2	123	benchmark
	8	Dry lining wall—gypsum fibre board	a4, a5, e5, a6, f2, g1	118	benchmark
	9	Dry lining wall—earth dry board	a10/b1	6	benchmark
			c1, d1, e3, f3, g1	124	
	10	Dry lining wall—wood fibre board	a10/b1	6	protection against overheating in summer, low emissions
			c1, d1, e6, f3, g1	124	adsorption capacity, acoustic protection
	11	Dry lining wall—earth block, dry stacked	a3, a12/b2	4	
			c1, d1, e3, h1, g1	258	
	15	Dry lining wall, Earth cellulose board	a9, d2, e2/b2, f3, g1	119	market innovation
	19	Non-load bearing, solid wall A—autoclaved aerated concrete	b4	2	cost efficient construction
			c1, d1, h2	105	
			c1 *(bonding of blocks)*	1	
	12	Non-load bearing, solid wall B—strawboards	a10/b1	6	market innovation
			c1, d1, e4	64	
	13	Non-load bearing, solid wall C—wood fibre board	a10/b1	6	market innovation
			c1, d1, f5, c2	126	
	14	Non-load bearing, solid wall D—wood fibre sandwich board with flax core	a10/b1	6	market innovation
			c1, d1, e7	75	
	16	Non-load bearing, solid wall E—reed blocks	a12/b2	3	cost efficient construction
			c1, d1, f6	105	
Internal insulation of external walls	17	Internal insulation external wall A—mineral insulation	a12/b2	3.0	high standard, refurbishment, mould resistant solution
			c1, d1, f7	35	
	18	Internal insulation external wall B—wood fibre insulation	a10/b1	6	high standard, refurbishment
			c1, d1, f4	65	

**Table 3:** Results of evaluation of investigated sample build-ups according to the AgBB scheme.

Sample No.	TVOC (µg/m^3^)			ΣSVOC (µg/m^3^)	Σ*R_i_*	Evaluation
	Assessable Compounds	Non-Assessable Compounds	Σ			
1	0	7 ^a^	7 ^b^	0 ^a^	0.0 ^c^	passed
2	0	91	91	53	0.0	passed
3	0	49 ^a^	49 ^b^	0 ^a^	0.0 ^c^	passed
4	0	66	66	46	0.0	passed
5	0	151 ^a^	151 ^b^	60 ^a^	0.0 ^c^	passed
6	0	28	28	11	0.0	passed
7	0	17 ^a^	17 ^b^	0 ^a^	0.0 ^c^	passed
8	0	16 ^a^	16 ^b^	10 ^a^	0.0 ^c^	passed
9	7	61	68	0	0.0	passed
10	17	76	93	0	0.0	passed
11	62	196	258	32	0.0	failed
12	0	7	7	1	0.0	passed
13	342	452	794	0	0.1	failed
14	92	41	133	9	0.0	passed
15	42	64	106	42	0.0	passed
16	17	79 ^a^	96 ^b^	10 ^a^	0.0 ^c^	passed
17	0	35 ^a^	35 ^b^	0 ^a^	0.0 ^c^	passed
18	7	89	96	0	0.0	passed
19	0	50	50	15	0.0	passed

^a^ Tests terminated after 10th sampling day. At this time, the value may not exceed 50 µg/m^3^; ^b^ tests terminated after 10th sampling day. At this time, the value may not exceed 500 µg/m^3^; ^c^ tests terminated after 10th sampling day. At this time, Σ*R_i_* must be ≤ 0.5.

## Data Availability

The data presented in this study are available on request from the corresponding author. At the time the project was carried out, there was no obligation to make the data publicly available.

## Supplementary Materials

The following are available online at https://www.mdpi.com/1996-1944/14/1/234/s1, Figure S1: Measured VOC and ΣSVOC concentrations: Samples no.: (a) 1; (b) 2; (c) 3; (d) 4; (e) 5; (f) 6; (g) 9; (h) 10; (i) 12; (j) 16; (k) 17; (l) 18; (m) 19.
