# Supplementary material for: Natural Building Materials for Interior Fitting and Refurbishment—What about Indoor Emissions?
Source: Materials (Basel). 2021 Jan 5;14(1):234. doi: 10.3390/ma14010234 (PMC7796468; doi:10.3390/ma14010234)
Supplement: Supplementary file 1 [file materials-14-00234-s001.pdf]

Supplementary materials

Natural building materials for interior fitting and refurbishment – What about indoor emissions?

Matthias Richter [1,*], Wolfgang Horn [1], Elevtheria Juritsch [1], Andrea Klinge [2,*], Leon Radeljic [2] and Oliver Jann [1]

[1] Materials and Air Pollutants Division, Bundesanstalt für Materialforschung und -prüfung (BAM), Unter den Eichen 44-46, 12203 Berlin, Germany; wolfgang.horn@bam.de (W.H.); ria.juritsch@bam.de (E.J.); oliver.jann@bam.de (O.J.)

[2] ZRS Architekten Ingenieure, Schlesische Straße 26, 10997 Berlin, Germany; radeljic@zrs.berlin (L.R.)

* Correspondence: matthias.richter@bam.de (M.R.); klinge@zrs.berlin (A.K.)

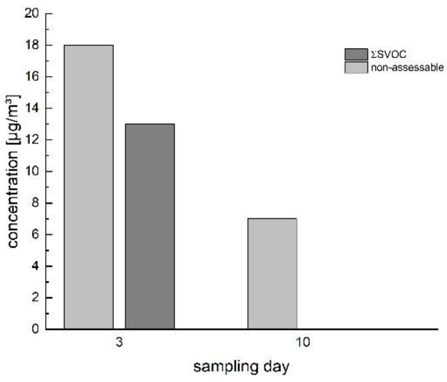

(a)

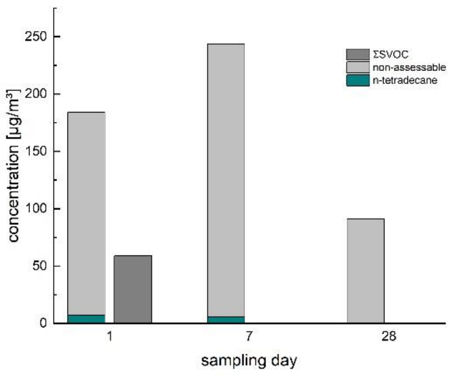

(b)

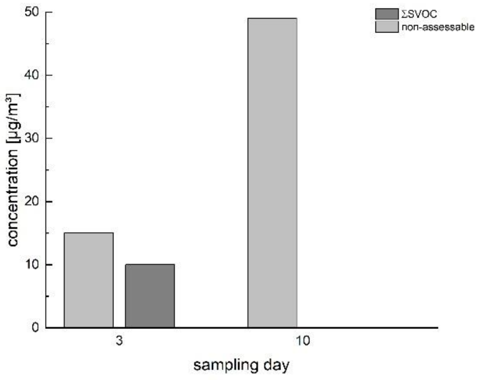

(c)

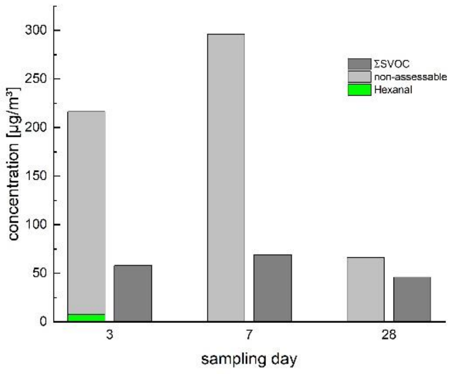

(d)

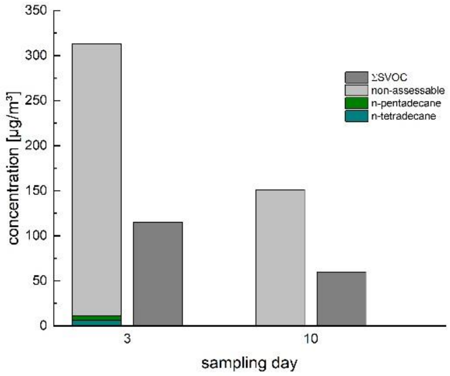
ΣSVOC
non-assessable
n-pentadecane
n-tetradecane
concentration [µg/m³]
sampling day

(e)

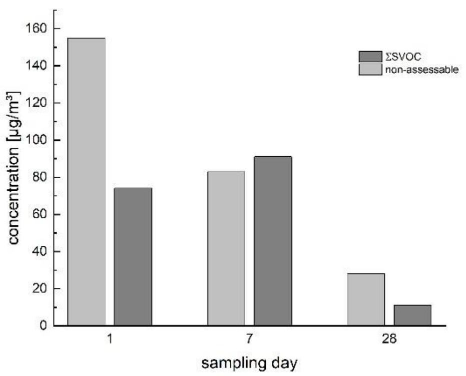
ΣSVOC
non-assessable
concentration [µg/m³]
sampling day

(f)

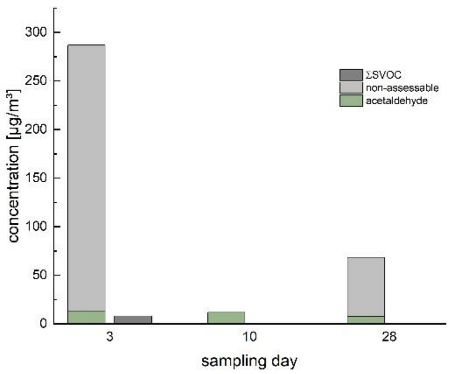
ΣSVOC
non-assessable
acetaldehyde
concentration [µg/m³]
sampling day

(g)

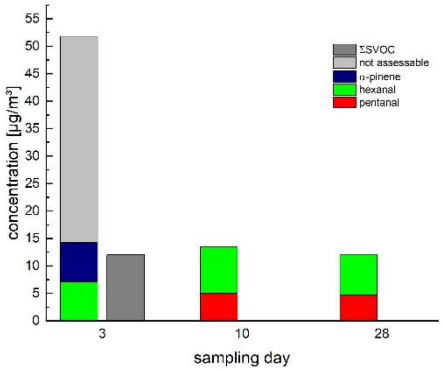
ΣSVOC
not assessable
α-pinene
hexanal
pentanal
concentration [µg/m³]
sampling day

(h)

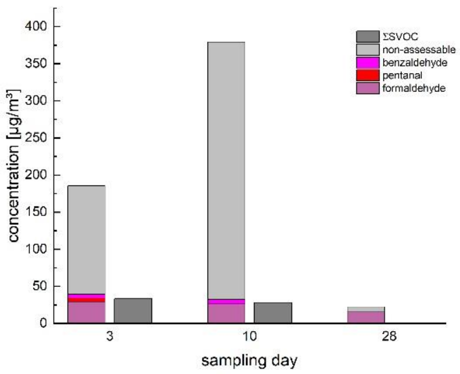
ΣSVOC
non-assessable
benzaldehyde
pentanal
formaldehyde
concentration [µg/m³]
sampling day

(i)

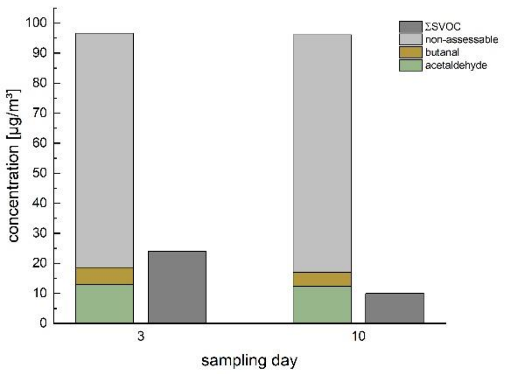
ΣSVOC
non-assessable
butanal
acetaldehyde
concentration [µg/m³]
sampling day

(j)

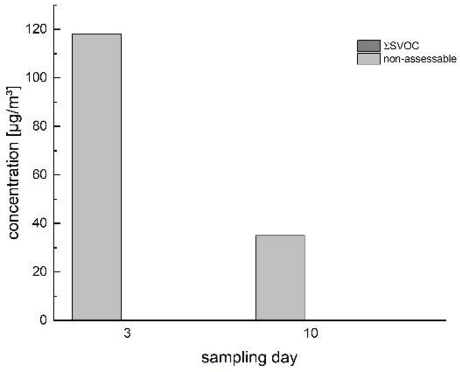

(k)

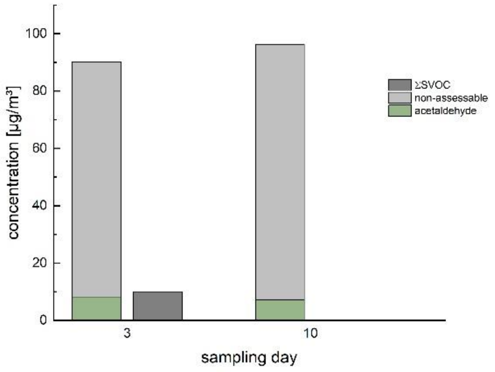

(l)

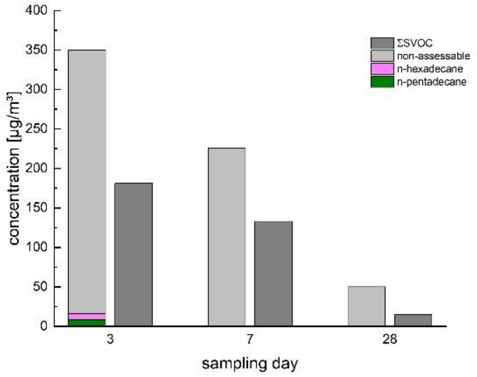

(m)

Figure S1. Measured VOC and ΣSVOC concentrations: Samples no.: (a) 1; (b) 2; (c) 3; (d) 4; (e) 5; (f) 6; (g) 9; (h) 10; (i) 12; (j) 16; (k) 17; (l) 18; (m) 19
